# Expression of Sex Hormone Receptors in Canine Osteosarcoma

**DOI:** 10.3390/vetsci9100524

**Published:** 2022-09-25

**Authors:** Kristyn N. Dilley, Alice Wong, Michael S. Kent, Michele A. Steffey, Clare E. Yellowley

**Affiliations:** 1 VCA Loomis Basin Veterinary Clinic, 3901 Sierra College Blvd, Loomis, CA 95650, USA; 2 Department of Anatomy, Physiology and Cell Biology, School of Veterinary Medicine, University of California Davis, 1285 Veterinary Medicine Drive, Davis, CA 95616, USA; 3 Department of Surgical and Radiological Sciences, School of Veterinary Medicine, University of California Davis, 1285 Veterinary Medicine Drive, Davis, CA 95616, USA

**Keywords:** osteosarcoma, estrogen, progesterone androgen, canine, hypoxia

## Abstract

**Simple Summary:**

Osteosarcoma is a bone tumor that arises from normal bone cells that become malignant. This is a disease that is relatively common in dogs and also affects humans. Dogs are a useful model for human osteosarcoma as they share many similarities including genetic changes, manner of spread and how they respond to treatment. In dogs, the tumors are frequently seen in large breeds and may be caused by environmental and genetic factors. Sex steroids have been shown to regulate bone metabolism directly and indirectly through receptors on bone. The aim of this study was to investigate the expression levels of sex hormone receptors in canine osteosarcoma tissue and cell lines. Our results demonstrate varying levels of receptors in tissue and a high expression of progesterone receptors in the canine osteosarcoma cell lines. Low oxygen levels further increased progesterone receptor expression. Lastly, estradiol decreased the expression of progesterone receptor in one of the cell lines, and progesterone decreased cell proliferation in the same cell line. Further investigation is recommended to determine whether or not sex steroid receptor levels and sensitivity to hormones are related.

**Abstract:**

Sex steroids regulate bone metabolism directly and indirectly through receptors on bone. Estrogen receptors (ER-∝, ER-β), progesterone receptor (PR), and androgen receptor (AR), have been previously identified on human osteosarcoma (OSA) cells, and are considered to influence tumor growth, but their expression and role in canine OSA is unknown. The aim of this study was to characterize sex hormone receptor expression levels in naturally occurring OSA tissue and in three canine OSA cell lines. The expression of ER-α, ER-β, PR, and AR was investigated using RT-PCR. PR expression levels were also quantified in OSA cells cultured under hypoxic conditions or in the presence of estradiol. The effects of progesterone on cell proliferation were quantified. Results demonstrated varying expression levels of these receptors in five OSA subtypes. OSA cell lines demonstrated high gene expression levels of PR and low gene expression levels of ER-α and ER-β and no gene expression of AR. PR expression was increased in OSA cells cultured under hypoxic conditions in a HIF-∝ independent manner. Interestingly, one cell line expressed very high levels of PR, expression of which decreased in response to estradiol. In addition, progesterone decreased OSA cell proliferation in this particular cell line. Further investigation of the role of sex steroids, particularly PR and its ligands, in regulation of canine OSA is recommended.

## 1. Introduction

Osteosarcoma (OSA) is an aggressive osteoid-producing neoplasm that accounts for the majority of primary bone tumors in the dog. OSA is most frequently seen in older large or giant breed dogs and may be histopathologically divided into five general subtypes: osteoblastic, chondroblastic, fibroblastic, telangictatic-mixed, or mixed [1]. The growth and regulation of cancer is affected by many variables, including hereditary predispositions, oncogene and tumor suppressor gene expression, the tumor microenvironment, immune system interactions, chronic inflammation and free radical production. Additionally, an effect of sex steroids on tumor occurrence and proliferation has been identified in some tumor histologies [2]. For example, the cellular proliferation of human breast cancer is stimulated by estrogen through oxidative damage and altered gene expression [2,3]. Similarly, sex steroids stimulate proliferation and growth of human OSA in vitro [4]. Some recent veterinary studies have suggested a correlation between neuter status and cancer prevalence although this remains controversial [5,6,7].

Sex steroids, particularly estrogen and androgen, directly regulate mammalian bone metabolism in both sexes. Androgens and estrogens influence growth hormone content and secretion from the pituitary gland, indirectly contributing to increases in growth and size [8]. ER∝ has been detected in osteoblasts, osteoclasts, and osteoblastic progenitor cells [9] and may regulate bone resorption and formation directly. Aromatizable androgens are transformed to estrogens by local aromatase present on bone cells [10]. This is consistent with concentration ratios of ER and AR identified on human osteoblasts [11]. The direct relationship between sex steroids and bone growth and metabolism is supported by the presence of sex steroid receptors on osteoblast-like cells [11,12].

The influence of sex steroids on the development and progression of cancer has been intensively studied. Elevated plasma and urinary levels of estrogen and testosterone (converted to estrogen by aromatase) are associated with an increased risk of breast cancer development in women [2]. In premenopausal women, low levels of progesterone are associated with an increased risk of breast cancer [2]. Furthermore, modulation of estrogen receptors using tamoxifen in breast cancer that expresses ER∝ or PR decreases the risk of recurrence [2]. Interplay between circulating hormones also plays an important role, for example, estradiol has been shown to upregulate the expression of progesterone receptors in human osteoblasts [13]. Even in primary tissues not involved in sexual reproduction, such as bladder, sex steroids were shown to play a role in cancer: AR knockout and androgen depletion in mice suppressed tumor growth in the bladder in vitro and in vivo [14].

Prior studies have confirmed the existence of sex steroid receptors in human OSA [4,15]. Like their tissue of origin, human osteoblast-like OSA cells (MG63) express AR, ER, and PR [4]. Additionally, estrogen, progesterone and DHT (dihydrotestosterone) all stimulated proliferation of MG63 human OSA cells in culture [4]. This suggests that circulating sex hormone milieu may influence the progression of OSA.

Hypoxia also plays an important role in carcinogenesis, initiation, the development of stem cell like phenotypes and cytotoxic drug resistance of tumor cells [16]. As tumors proliferate and obstruct vascular flow, induce abnormal vasculature and/or outgrow their vascular supply, oxygen availability is compromised and cells adapt to this low oxygen tension by activating several pathways to ensure survival [16]. Such pathways include upregulation of genes that promote energy conservation, cell survival and angiogenesis, which support tumor growth; pathways which are commonly mediated via the HIF-1 transcription factor. Additionally, HIF-1 initiates the transcription of over 100 genes involved in drug resistance and cellular proliferation [16,17]. Transcriptional targets include drug transport pathways such as p-glycoprotein or topoisomerase II, oncogenic growth factors such as TGF-β3, and angiogenic growth factors such as VEGF [16,17]. Correlations between the HIF-1 pathway and sex steroid receptors have been studied in humans. In human prostate cancer, HIF-1 signaling has been shown to limit AR gene expression and thus lead to resistance to AR antagonist drug therapy [18]. In a study of canine breast cancer, elevated HIF-1 expression in the tumor was significantly correlated with negative ERα expression [19]. However, the effects of hypoxia and HIF transcription factors on steroid expression has not yet been studied in canine OSA.

The goal of this study was to characterize sex hormone receptor expression levels in naturally occurring OSA tissue samples characterized by a variety of histologic subtypes and in three canine OSA cell lines.

## 2. Materials and Methods

### 2.1. Canine OSA Samples

Twelve samples of canine OSA tissue were taken from formalin-fixed paraffin-embedded (FFPE) tissue biopsies from clinical patients at the University of California Davis Veterinary Medical Teaching Hospital. Tissues were sectioned from banked OSA samples biopsied between the years of 2006–2014 from large-breed male castrated and intact dogs, Table 1 A single pathologist evaluated all samples to classify the OSAs and ensure quality representative samples of neoplastic growth. Normal cortical bone was similarly derived from the biopsy bank at the University of California Davis Veterinary Medical Teaching Hospital. Normal testes and ovaries were obtained from animals undergoing elective neutering procedures were derived from surgical waste tissues and were immediately snap frozen and stored at 0oC in phosphate-buffered saline (PBS) and RNAlater. All biological samples were obtained via client consent. 

### 2.2. Cell Culture

In a previous report, we describe the isolation of cells from naturally occurring OSA in three individual dogs, which were grown in vitro and cloned to produce three cell lines, #529, #484, and #617 [20,21], Table 2. Cell line #484 was shown to express osteocalcin, osteonectin and alkaline phosphatase and induced tumors when injected into mice that resembled histologically the parent canine tumor (osteoblastic) from which the cells were derived [20].

All cell lines were cultured at a density of 10,000 cells/cm^2^ in 10 cm Petri dishes in Minimum Essential Medium, alpha modification (α-MEM), supplemented with 10% fetal bovine serum (FBS) and 1% penicillin and streptomycin. Cells were maintained in a standard humidified incubator at 37 °C with 95% ambient air and 5% CO_2_ atmosphere. Cells were routinely subcultured with PBS and 0.05% trypsin/EDTA. For hypoxic culture, cells were cultured in hypoxic media (∝-MEM supplemented with 10% FBS and 1% penicillin and streptomycin, preconditioned at 1% oxygen overnight) for 24 h in a humidified incubator at 37 °C with 5% CO_2_ and O_2_ tension reduced to 1% with supplemental nitrogen. Some cells were cultured in standard media with 100 μg/mL desferrioxamine (DFO) to induce HIF stabilization and cultured under standard conditions. As prior studies on human osteoblastic cells have shown upregulation of progesterone receptors in response to estrogen via both ER isoforms [12], the effect of estradiol on PR expression in OSA cells was studied. Linearly increasing concentrations (0, 10, 100, 1000 nM) of estradiol were added to cell cultured for 24 h.

### 2.3. Isolation of RNA

RNA was extracted from canine tissue using a standard RNA isolation protocol for paraffin-embedded samples (PureLink™ FFPE Total RNA Isolation kit, Invitrogen Corporation, Carlsbad, CA, USA). RNA was isolated from cell lines (RNeasy Mini Kit, Qiagen, Hilden, Germany). Fresh tissue control RNA was isolated using lysis buffer and β-mercaptoethanol, before washing in PBS and homogenization with liquid nitrogen (LN_2_). RNA concentrations and 260/280 ratios were determined using spectrophotometry (Thermo Scientific Nanodrop 1000, Thermo Fisher Scientific Inc., Waltham, MA, USA). If needed, samples were diluted with sterile H_2_O to produce RNA concentrations less than 500 ng/μL. Total RNA was subsequently stored in a −80 °C freezer.

### 2.4. Reverse Transcription and qRT-PCR

0.2–1 mg of total RNA from all canine tissues and cell lines was reverse-transcribed (QuantiTect reverse transcription kit, Qiagen, Hilden, Germany) following the manufacturer’s protocol and cDNA was stored in −40 °C freezer. qRT-PCR was performed to determine the expression levels of steroid receptors. PCR primers are specific to canine genes and were purchased from Applied Biosystems. A PCR kit (QuantiFast Probe, Qiagen, Hilden, Germany) was used to amplify the following genes: estrogen receptor alpha (ERα) [Cf02624846_m1], estrogen receptor beta (Erβ) [Cf02624849_m1], progesterone receptor (PR) [Cf02623807_m1], androgen receptor (AR) [Cf02623880_m1], and β-2 microglobulin (B2M) housekeeping gene [Cf02659077_m1]. PCR products were amplified under the following conditions: 95 °C for 3 min, followed by 40 cycles at 95 °C for 3 s and 60 °C for 30 s. Gene expression was normalized to the housekeeping gene (ΔCT) and evaluated in comparison to control expression levels.

### 2.5. PR and Cell Proliferation Assays

Cell lines were serum-starved via suspension in FBS-free medium to produce stock concentrations at a density of 5000 cells/mL. 200 μL of this solution was placed into each well of a 72-well plate and cultured for 48 h. After the addition of 0, 100, and 1000 nM progesterone for a further 24 h, 20 μL of Alamar Blue (Ex 530 nm, Em 590 nm) was added to each well and incubated for 24 h. Cell proliferation was measured using a fluorescence plate reader.

### 2.6. Statistical Analysis

Data were analyzed by one-way ANOVA (Graph Pad Prism). Post hoc comparisons with control values were performed using Bonferroni’s multiple comparisons tests. Statistical significance was considered at *p* < 0.05.

## 3. Results

### 3.1. Gene Expression in Canine OSA Tissue and OSA Cell Lines

**Figure 1:**
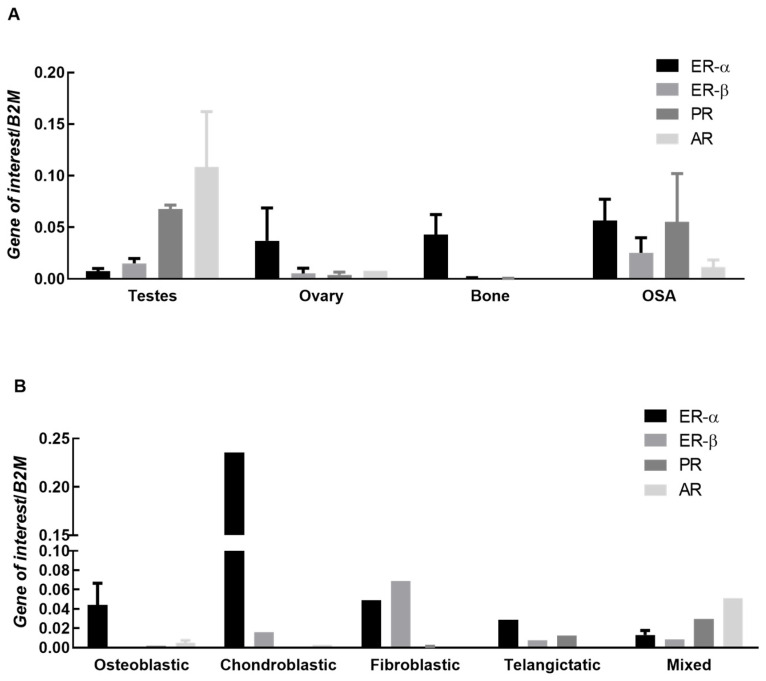
Expression of ERα, ERβ, PR and AR in canine OSA tissue and subtypes. (**A**) Expression of estrogen receptor α (ER-∝), estrogen receptor β (ER-β), progesterone receptor (PR), and androgen receptor (AR) genes in ovary and testes (controls), normal bone, and OSA tissue. Bars represent mean 2^−∆Ct^ ± SEM, with expression normalized to B2M. Sample size controls; n = 3 testes, 3 ovaries and 3 bone (all biological replicates). Sample size OSA tissue samples n = 12; (**B**) Expression of estrogen receptor α (ER-∝), estrogen receptor β (ER-β), progesterone receptor (PR), and androgen receptor (AR) genes in OSA tissue. Bars represent mean 2^−∆Ct^ ± SEM, with expression normalized to B2M. Sample size n = 4 osteoblastic, 1 chondroblastic, 2 fibroblastic, 2 telangictatic and 3 mixed (all biological replicates).

RT-PCR was completed on 12 OSA biopsies and compared against several positive controls: bone, ovary, and testes. Sample size was limited by the qualifications of the study, biopsies available in the banked pathology database, and RNA integrity in FFPE tissue. Useable samples data were combined and expression patterns compared to controls, Figure 1A. Samples were further subdivided into osteoblastic, fibroblastic, chondroblastic, mixed, and telangictatic samples, Figure 1B. Combined data demonstrated expression of all receptors in OSA tissue, Figure 1A. The AR receptor was poorly expressed in all OSA subtypes with the exception of mixed, Figure 1B. ER-∝ was the most highly expressed receptor in osteoblastic OSA tissue with no detectable expression of ER-β and PR, and low expression of AR, Figure 1B. Chondroblastic OSA tissue demonstrated very high levels of ER-∝, low levels of ER-β, while PR was undetectable and AR expressed at very low levels, though it should be noted that there was only one sample available for assessment, Figure 1B. Fibroblastic OSA tissue expressed both ER-∝ and ER-β, very low levels of PR, and AR was not detected, Figure 1B. Telangictatic OSA tissue expressed all receptors except AR, Figure 1B. Mixed OSA samples expressed all receptors with AR being the most highly expressed of the four, Figure 1B.

**Figure 2:**
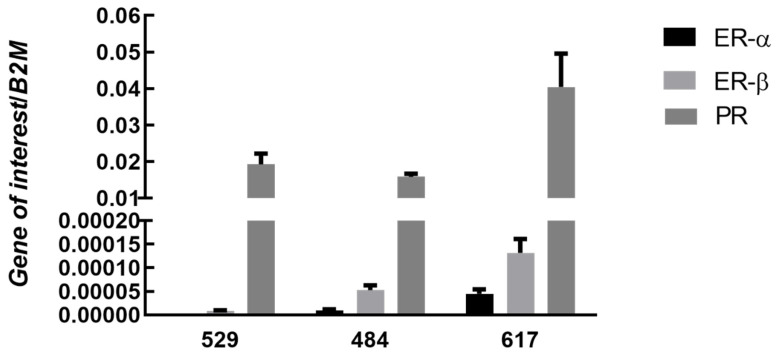
Expression of ERα, ERβ, and PR in canine OSA cell lines. Bars represent mean sex steroid receptor expression 2^−∆Ct^ ± SEM, with expression normalized to B2M in three osteosarcoma cell lines 529 (n = 7), 484 (n = 7), 617 (n = 7) (technical replicates). Note: there was no detectable expression of AR in any cell line.

Similar to OSA tissue, steroid receptors were expressed variably in each OSA cell line, Figure 2. In all cell lines, the progesterone receptor (PR) showed the highest level of expression. Estrogen receptors were expressed at low levels in all three cell lines and AR was not expressed.

### 3.2. Effect of Hypoxia on PR Gene Expression

**Figure 3:**
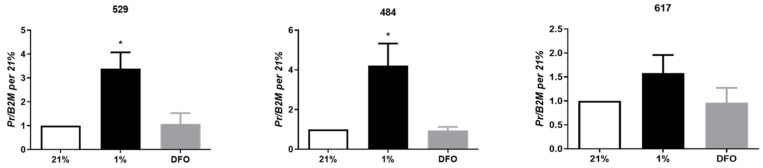
Hypoxic regulation of PR. Expression of PR in OSA cells (cell lines 529, 484 and 617) at 24 h of exposure to 21% oxygen, 1% oxygen or desferrioxamine (DFO). Bars represent mean 2^−^^ΔCt^ ± SEM, with expression normalized to B2M and 21% O_2_, n = 3 technical replicates for each individual cell line. * indicates statistically significant difference compared to expression under 21% oxygen and DFO at 24 h, *p*-value < 0.05.

Examination of the effect of hypoxia (1% oxygen) and desferrioxamine (DFO), a hypoxia mimetic which promotes the accumulation of HIF transcription factors, on OSA cells demonstrated that PR expression was significantly elevated in response to 1% oxygen at 24 h in cell lines 529 and 484 (Figure 3); there was a trend for increased PR expression cell line 617 (*p* < 0.36). DFO had no effect on PR expression, suggesting that hypoxia-induced PR expression occurs via a HIF independent mechanism in OSA cells.

### 3.3. Effects of Exogenous Estradiol on PR Gene Expression

**Figure 4:**
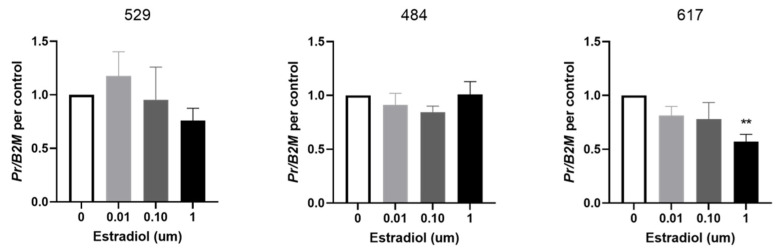
Effect of estrogen on PR expression in OSA cells. Expression of progesterone receptor (PR) in OSA cells (cell lines 529, 484 and 617) after 24 h of treatment with estradiol at 0 µM (vehicle control), 0.01 µM, 0.1 µM, and 1 µM. Bars represent mean PR expression 2^−∆Ct^ ± SEM, with expression normalized to B2M and control, n = 4–6 technical replicates for each individual cell line. ** indicates statistically significant difference from 0 µM, *p* < 0.01.

PR expression in cell line 617 was significantly decreased in response to 1 µM of estradiol at 24 h compared to control (Figure 4). Estradiol had no effects on PR expression in cell lines 529 and 484.

### 3.4. Cell Proliferation in Response to Exogenous Progesterone

**Figure 5:**
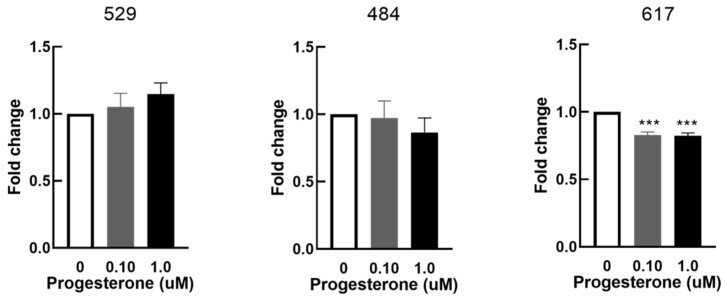
Effect of progesterone on cellular proliferation in vitro. Cellular proliferation in OSA cells (cell lines 529, 484 and 617) after 48 h of treatment with progesterone at 0 µM (vehicle control), 0.1 µM, and 1 µM. Bars represent mean fold changes in Alamar blue fluorescence normalized to vehicle controls, n = 4 technical replicates for each individual cell line. *** indicates statistically significant difference from 0 µM, *p* <0.001.

OSA cell proliferation was significantly decreased in cell line 617 in response to 0.1 µM and 1 µM progesterone at 48 h when compared to control (0 µM of progesterone) (Figure 5). Progesterone had no effects on cell proliferation in cell lines 529 and 484.

## 4. Discussion

Dogs have been described as excellent models of human osteosarcoma given the striking similarities in presentation of the disease in canines and humans, including risk factors, location, disease etiology and metastatic profile [22,23]. Expression levels of markers associated with human OSA including alkaline phosphatase, desmin, bone morphogenetic protein 4 and runt related transcription factor 2 were described in spontaneously arising canine osteosarcoma [24]. Given the similarities in expression, canine OSA was suggested to be a useful model of human OSA [24]. Derivation of canine OSA cell lines from spontaneously occurring canine osteosarcoma can therefore provide novel in vitro and in vivo models to study both canine and human OSA disease. Indeed cell line #484 utilized in this study is known to regenerate an OSA tumor with histology similar to the parent tumor from which the line was derived [20].

Multiple studies have shown an association between incidence of OSA and involvement of sex steroids by gonadal exposure or a lack thereof in both humans and animal models [5,6,25]. Herein we demonstrate expression of estrogen receptor α (ER-∝), estrogen receptor β (ER-β) and progesterone receptor (PR) in three OSA cell lines. These same receptors were expressed in all OSA tissue samples though at varying levels. The androgen receptor (AR) was not expressed in OSA cells lines and was poorly expressed in 4 of the five subtypes of OSA tissue. Interestingly, in an immunohistochemical study of human OSA tissues, 24 of 28 cases demonstrated immunoreactivity for PR, 23 of 28 demonstrated ER-β, 8 of 28 cases demonstrated AR and no cases demonstrated ER-∝ [4]. This is very similar to the expression profile of steroid receptors we report for canine OSA cells lines. High expression levels of PR and ER-β have also been described in the human OSA line, MG-63 [4]. To our knowledge, we identify for the first time the presence of sex steroid receptors on canine OSA tissue and on canine OSA cell lines. Further, this expression profile appears to be consistent with that described for human OSA cells and tissue.

The progesterone receptor was the most highly expressed steroid receptor in canine OSA cells. Levels of PR in OSA tissues were high and increased relative to those expressed in normal bone, Figure 1A. Progesterone is known to be involved in bone remodeling and trabecular bone density [13], and PR expression has been detected in high levels on human OSA lines and osteoblasts [4]. The consistent presence of PR to a high degree in canine OSA in combination with the effects of its direct ligand on bone regulation suggests potential for a role in tumorigenesis. No androgen receptors were detected on any of the three cell lines. Estrogen isoforms are variably expressed throughout different periods of osteoblastic differentiation [26] and differences in individual OSA differentiation might account for varying expression levels. Our data suggests that expression levels of ER-∝, ER-β, PR, and AR may differ between OSA subtypes, though a limitation is our low sample numbers. Of note, high levels of ER-∝ were expressed by the chondroblastic sample, and further studies are needed to verify these findings and to determine the significance. Although histopathologic subtype has no significant association with tumor grade or metastatic status [27], each may demonstrate unique receptor expression and therefore, response to exogenous hormonal stimulation.

We demonstrate hypoxic regulation of PR expression in OSA cells which is independent of the HIF pathway. Hypoxia, a common feature of tumors that have outgrown their blood supply, is significant to tumor biology through its ability to suppress apoptosis, promote tumor progression, and induce drug-resistance [16]. HIF-1, which is stabilized under hypoxic conditions, is the major transcription factor responsible for the cellular adaptation to hypoxia [16]. PR expression levels did not change in OSA cells treated with the hypoxia mimetic DFO suggesting that the influence of hypoxia on PR may not require HIF signaling. This points to the existence of a HIF independent mechanism of hypoxia-driven gene regulation and potential drug resistance. Hypoxia plays an important role in tumor growth by regulating the expression of genes that can not only modulate drug-resistance, but cell survival and angiogenesis [16]. Adamski et al. demonstrated that hypoxia selected for p53 deficient cells in oncogenically transformed cells in p53 dependent apoptosis, decreasing levels of the tumor suppressor gene to ensure OSA survival and progression [16]. Whether hypoxic regulation of PR in OSA contributes to OSA growth and survival is unknown.

Our results established the presence of progesterone and estrogen receptors on three canine OSA cell lines, the most significant of which was PR expression. Previous studies on human osteoblastic cells demonstrated an upregulation of PR in response to estrogen [13]. Estrogen signaling through both ER-α and ER-β were determined to induce PR expression in fetal osteoblast lines, concluding that PR may be present in all osteoblasts that express ER-α or ER-β [13]. We aimed to investigate a similar effect in canine OSA cells, and subsequently studied the effects of the addition of estradiol on cells to determine estrogenic influence on PR. However, in our study, OSA cells either did not respond (cell lines #529 and 484) or significantly decreased the expression of PR after the addition of estradiol (cell line #617). The reason for this variation in response is unclear, but could be due to the supraphysiologic concentrations used relative to normal canine hormone concentrations [28].

Prior studies on human OSA cell line MG-63 also showed significant proliferation secondary to the addition of progesterone [4]. Furthermore, a PR blocker showed a significant suppression of proliferation of MG-63 cells. They therefore suggested that steroid antagonists have the potential to be used as suppressors of neoplastic proliferation in human OSA. We similarly wanted to characterize this relationship in canine OSA cells. Proliferation assays demonstrated either no response (cell lines #529 and #484) or a significant decrease in the proliferation of canine OSA cells in response to the addition of exogenous progesterone at concentrations at or greater than 0.1 µM (cell line #617). The inconsistency in results with our study could again be due to supraphysiologic levels of progesterone in comparison to normal canine levels utilized in our assay [28], or variation in OSA lines and species. Interestingly cell line #617 which showed an estradiol-induced decrease in PR expression and a progesterone-stimulated decrease in proliferation had the highest expression of the progesterone receptor. Despite the inconsistency seen between our results and those of previous studies [4], these findings do demonstrate that in cells with high expression levels of PR, an estrogenic effect on PR expression and a potential for suppression of canine OSA cell proliferation with progesterone.

Prior studies examining the effect of neutering on cancerous growth have shown twice the disease occurrence of OSA in neutered dogs [5,6]. As elective neutering disturbs the concentrations of circulating sex hormones in the body by removing their primary source, this increased prevalence suggests that tumor incidence and growth may be related to the timing or duration of gonadal hormone exposure or sex hormone concentration [6]. Although these studies have pointed to a correlation between neutering and neoplastic growth, this remains a debated and controversial topic in the literature. One study demonstrated that there is no effect of neutering on lymphosarcoma, mast cell tumor, and hemangiosarcoma occurrence in male Golden Retrievers and Labradors [29]. However, Hoffman et al. suggest that sterilization increases the risk of death by neoplasia, even when analyzed in differing age categories [30]. A study on the incidence of OSA in Rottweilers as a human model demonstrated a strong inverse dose–response relationship between risk of bone sarcoma and lifetime exposure to gonadal steroids as determined by months intact [25].

## 5. Conclusions

Three canine OSA cell lines demonstrated high gene expression levels of PR, low gene expression levels of ER-α and ER-β and no gene expression of AR. PR expression was increased in OSA cells cultured under hypoxic conditions in a HIF-1 independent manner. Our data show that sex steroid hormone receptors exist in canine OSA tissue and therefore demonstrate the potential for modulation by circulating hormones, particularly by PR and its ligands. However, a potential relationship between steroid receptor expression levels and hormone sensitivity is warranted.

## Figures and Tables

**Table 1:** Age, sex and breed for canine OSA tissue. MC male castrated, MI male intact.

	Age (Yr)	Sex	Breed
Chondroblastic	11	MC	Labrador
Fibroblastic	4	MC	Saint Bernard
Fibroblastic	10	MC	Labrador Retriever
Mixed	11	MC	Greyhound
Mixed	7	MI	German Shorthair Pointer
Mixed	12	MC	Labrador Retriever mix
Osteoblastic	5	MI	Great Dane
Osteoblastic	10	MI	Labrador
Osteoblastic	5	MC	Golden Retriever
Osteoblastic	5	MC	Anatolian Shepherd
Telangictatic	8	MI	Irish Wolfhound
Telangictatic	10	MC	Labrador Retriever mix

**Table 2:** Case/clone number, sex, age and breed used to derive cell lines.

Cell line/ Case/Clone #	Sex	Age Yrs	Breed
529	M	8	Doberman
484	M	8	Mixed Newfoundland/Labrador
617	M	5	Neapolitan Mastiff

## Data Availability

Not applicable.
